# Effect of Annealing Time and Temperature Parameters on the Microstructure, Hardness, and Strain-Hardening Coefficients of 42CrMo4 Steel

**DOI:** 10.3390/ma13092022

**Published:** 2020-04-26

**Authors:** Mirosław Szala, Grzegorz Winiarski, Łukasz Wójcik, Tomasz Bulzak

**Affiliations:** 1 Department of Materials Engineering, Faculty of Mechanical Engineering, Lublin University of Technology, Nadbystrzycka 36D, 20-618 Lublin, Poland; 2 Department of Computer Modelling and Metal Forming Technologies, Faculty of Mechanical Engineering, Lublin University of Technology, Nadbystrzycka 36D, 20-618 Lublin, Poland

**Keywords:** steel, microstructure, annealing, upsetting, hardness, strain-hardening

## Abstract

The study presents the effect of annealing process parameters on the microstructure, hardness, and strain-hardening coefficients, that is, the strength coefficient *c* and the strain-hardening exponent *n*, of 42CrMo4 steel. Seven selected annealing time–temperature schemes are examined for superior steel formability in cold metal forming conditions. The *c* and *n* coefficients are first determined in experimental upsetting of annealed samples and then used in FEM (finite element method) simulations of the upsetting process. The results demonstrate that the strain-hardening coefficients (*c* and *n*) depend on the employed annealing scheme. Compared with the as-received sample, the annealing process reduces the true stress and effectively decrease the hardness of 42CrMo4 steel; improves microstructural spheroidization; and, consequently, facilitates deformability of this material. The annealing schemes, relying on heating the material to 750 °C and its subsequent slow cooling, lead to the highest decrease in hardness ranging from 162 to 168 HV. The results obtained with the SEM-EDS (scanning electron microscopy-energy dispersive spectrometer), LOM (light optical microscopy), and XRD (X-ray diffraction) methods lead to the conclusion that the employed heat treatment schemes cause the initial ferritic-pearlitic microstructure to develop granular and semi-lamellar precipitation of cementite enriched with Mo and Cr in the ferrite matrix. In addition, the annealing process affects the growth of α-Fe grains. The highest cold hardening rate, and thus formability, is obtained for the annealing scheme producing the lowest hardness. The results of FEM simulations are positively validated by experimental results. The obtained results are crucial for further numerical simulations and experimental research connected with developing new cold metal forming methods for producing parts made of 42CrMo4 steel.

## 1. Introduction

Low-alloy structural steels are widely used in mechanical, automotive, mining, and machine building industries and undergo shaping by metal forming, machining, welding, surfacing, and many other techniques [1,2,3,4,5]. Particularly, the structural steel grade 42CrMo4 (AISI 4140) offers the most versatile applications and is one of the most universal grades dedicated to heat treatment and plastic deformation [1,2,6,7,8]. However, to facilitate its deformability in cold metal forming processes, this steel requires a special heat treatment—annealing. Although the general definition of the process is well-known and describes “any heating and cooling operation that is usually applied to induce softening” [9], accurate selection of the process parameters for treating a specific steel grade seems to be a complex procedure. Annealing refers to altering mechanical or physical proprieties of a material by producing a definite microstructure. Unfortunately, annealing time–temperature parameters, like in other heat treatments, are not only affected by the chemical composition of steel, but they also depend on the combination of metal treatment history (primary microstructure), initial plastic deformation, part dimensions, and many other factors affecting the properties of heat-treated 42CrMo4 steel [1,8,9,10,11,12]. Moreover, specific time–temperature conditions of the annealing process should be selected individually depending on the part being treated. Despite the fact that the literature of the subject [8,9] reports general parameters of annealing, still, the best solution for obtaining superior deformability of specific steels is to conduct an experimental investigation of time–temperature parameters of this heat treatment.

It is generally believed that the examination of hardness and microstructure is the best method for evaluating the quality of a conducted heat treatment. Nevertheless, in industrial conditions, the particularly time-effective measurement of hardness is employed to examine the softening of steel before subjecting the treated steel to metal forming processes. Although, in many cases, light optical microscopy (LOM) and scanning electron microscopy (SEM) give accurate results of metallographic examination [7,13,14,15], electron back-scattered diffraction (EBSD) [16,17], transmission electron microscopy (TEM) [1,18], and X-ray diffraction (XRD) [19,20,21] seem to be much more powerful tools for evaluating microstructure development owing to plastic deformation or heat treatment. In [20], the authors employ XRD to study the hardening and tempering behaviour of the En24 steel (ISO equivalent 34CrNiMo6) via X-ray peak profile analysis. In addition, Bouras et al. [21] claim that the XRD peak broadening is in a direct relation with the structural anisotropy and heterogeneity in the rolling plane during deformation by deep drawing of mild soft steel.

Additionally, many researchers have stressed the importance of calculating strain-hardening coefficients such as a strength coefficient, *c*, and a strain-hardening exponent, *n*. They are particularly essential when selecting heat treatment technological parameters or elaborating metal forming technology for steel [22,23], aluminium alloys [24,25], magnesium alloys [26,27], pure copper [28], powder metallurgy materials [29,30], or plasticine [31,32], or even MMC (metal matrix composite) materials [33,34]. Therefore, this paper investigates the effect of annealing on the strain-hardening parameters, and the *c* and *n* coefficients are determined and can be used in numerical simulations and experimental tests of 42CrMo4 steel components dedicated for metal forming [35,36].

Although 42CrMo4 steel (AISI 4140) is a popular machine-building material, the literature recommends different annealing procedures for softening the material and facilitating its cold working, not to mention the fact that the literature of the subject gives various annealing parameters [6,8,9,15,37]. In addition, a very important factor in industrial conditions is to obtain the highest steel deformability in an optimal time of the annealing process. From this point of view, it is worth determining the accurate annealing time and temperature parameters of 4140 steel. Moreover, FEM simulations of the cold forming process effectively shorten the time consumed by the design of a metal forming process. However, to obtain accurate simulation results of the annealed steel, the pre-calculation of strain-hardening coefficients is required.

This research focuses on studying the effect of 42CrMo4 steel annealing parameters on the microstructure development as well as the hardness and strain-hardening coefficients of the material. Thus, in this work, seven annealing schemes with different times and temperatures were studied to identify the optimum cold formability of 42CrMo4 steel. The treatments were selected for the lowest hardness and superior microstructural deformability, and subsequently verified by cold upsetting tests. The obtained findings are essential for future research on the design of new cold metal forming techniques for producing 42CrMo4 steel parts.

## 2. Materials and Methods

### 2.1. Investigated Steel and Heat Treatment

**Figure 1:**
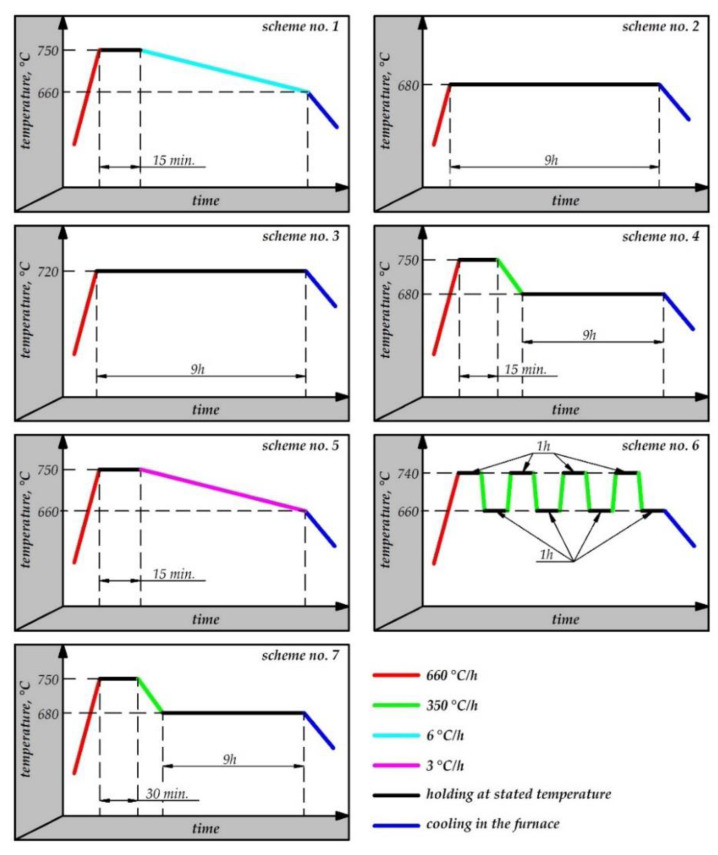
Time vs. temperature schemes of the annealing process.

The study was performed on low-alloy structural steel grade 42CrMo4 (AISI 4140); its chemical composition and mechanical properties given in Table 1. Cylindrical samples with the dimensions ø15 mm × 18 mm were heat-treated according to seven different annealing schemes. The employed annealing schemes are shown in Figure 1, and they were designed according to the literature data [6,8,9,15,37] and engineering practice. Schemes consist of treating the steel using different time and temperature parameters around the A_1_ temperature with different cooling rates. Generally, the practical goal of the experiment was to select the most time-effective annealing process, while the scientific aim was to study how the different time and temperature parameters of annealing affect the microstructure, hardness, and strain-hardening coefficients. Overall, the main objective of the annealing treatment was to obtain the highest deformability (steel softening) in the shortest time-consuming process. The sample in as-received conditions was denoted as 0, while other heat-treated samples were denoted in compliance with the scheme numbers from 1 to 7; see Figure 1. The treatment schemes consisted in first heating the material to a temperature above A_c1_, and then either cooling it very slowly in a furnace (schemes no. 1 and 5) or maintaining it at a temperature just below A_r1_ (schemes no. 4 and 7), or prolonged holding of the material at the A_c1_ temperature (schemes no. 2 and 3) followed by alternate heating and cooling of the material at temperatures that are just below A_r1_ and just above A_c1_. After that, hardness and microstructure were comparatively analysed and examined in relation to the findings obtained in upsetting (compression test).

### 2.2. Characterization of the Annealed and Deformed Samples—Microstructure and Hardness

The samples were examined as received and after annealing, before and after the upsetting test. The structure was investigated on the polished and etched transverse section of the samples by light optical microscopy (LOM, Nikon Eclipse MA200) (Tokyo, Japan) and scanning electron microscopy (SEM, Phenom ProX Desktop SEM, Phenom World (Waltham, MA, USA)) equipped with energy dispersive spectrometer (EDS). Moreover, the phase structure was evaluated qualitatively and quantitatively by X-ray dispersive diffraction (XRD) using the XTRa ARL X-ray diffractometer (manufactured by Thermo Fisher Scientific, Massachusetts, Waltham, MA, USA) and the parameters described in [38,39]. Vickers hardness was measured before and after the heat treatment and compared with the hardness results of the upset samples. To determine the load effect, both Vickers HV 10 macro-hardness and Vickers HV 0.3 micro-hardness indentations were made on the flat surface of the cylindrical samples. Moreover, after upsetting, the HV 0.3 hardness was measured on the transverse section of the samples, and, to ensure statistical accuracy, at least 16 indentations were made. Finally, the cold hardening ratio of every sample was calculated as a ratio of the upset sample hardness to the initial (annealed) sample hardness. The effects of hardness on the strain hardening coefficients were analysed.

### 2.3. Comparison of the Experimental and FEM Results of Upsetting 

The FEM (finite element method) simulation of the upsetting process was followed by experimental tests. Prior to modelling, it was necessary to experimentally determine the strain hardening coefficients *c* and *n* (*c* denotes the strength coefficient and *n* denotes the strain hardening exponent). The determined *c* and *n* values are crucial for accurate modelling of annealed 42CrMo4 steel. These parameters were determined during the upsetting test. For that reason, the flow cures of the as-received and annealed samples were estimated by upsetting testing. The samples made of 42CrMo4 steel with the dimensions ø15 mm × 18 mm (d_0_ × h_o_*)* were compressed with a strain rate of 0.2 s^−1^, using the static testing machine Instron 1000HDX (Instron, Norwood, MA, Canada). The tests were carried out in ambient temperature until the upsetting force limit of 800 kN was reached. Three upsetting tests, one for each annealing scheme, were performed. The instantaneous strain (ε_i_) and flow stress (σ_pi_) were determined using Relationship (1).

(1) $$\{\begin{array}{c} \varepsilon_{i}=\ln\frac{h_{0}}{h_{i}}\\ \sigma_{\mathrm{pi}}=\frac{4\times F_{i}\times h_{i}}{\pi \times d_{0}^{2}\times h_{0}} \end{array}$$

where h_0_ and d_0_ are the initial height and diameter of the samples, respectively; and h_i_ and F_i_ are the instantaneous height of the sample and its corresponding instantaneous upsetting force, respectively.

The flow curves were described by constitutive equations expressed with Relationship (2). To determine the coefficients *c* and *n* in the equation, the objective function F_σ_ (described by Equation (3)) was determined, and then the values of c and n for which this function would reach the minimum value were determined.

(2) $$\sigma_{p}=c\times \varepsilon^{n}$$

(3) $$F_{\sigma }=\overset{n}{\underset{i=1}{\sum }}\left[{\left(\sigma_{\mathrm{pi}-1}-\sigma_{p}\right)}^{2}+{\left(\sigma_{\mathrm{pi}-2}-\sigma_{p}\right)}^{2}+{\left(\sigma_{\mathrm{pi}-3}-\sigma_{p}\right)}^{2}\right]\Rightarrow \min$$

where σ_pi-1_, σ_pi-2_, σ_pi-3_ are the flow stresses at *i*-th measuring point determined by means of Relationship (1) for individual samples in the same heat treatment condition; and σ_p_ is the flow stress described by constitutive Equation (2).

The upsetting process was modelled under the axisymmetric state of strain using the DEFORM 2D/3D commercial software (Scientific Forming Technologies Corporation, Columbus, OH 43235, USA; version 11.0). It was performed on a 42CrMo4 steel rod, the material model of which was obtained from the material database library of the simulation software, while the stress hardening coefficients were applied based on the upsetting experimental results determined in the present study. Forging shape and force parameters, as well as stress and strain distributions, among others, are shown in the results. The metal forming simulation parameters are described in detail in [35,36] in relation to the cold metal forming of 42CrMo4 steel parts. Finally, the effects of computer modelling were validated by experimental upsetting of samples.

## 3. Results and Discussion

### 3.1. Heat Treatment Effect on 42CrMo4 Steel Properties

#### 3.1.1. Microstructures’ Development Owing to Heat Treatment

**Figure 2:**
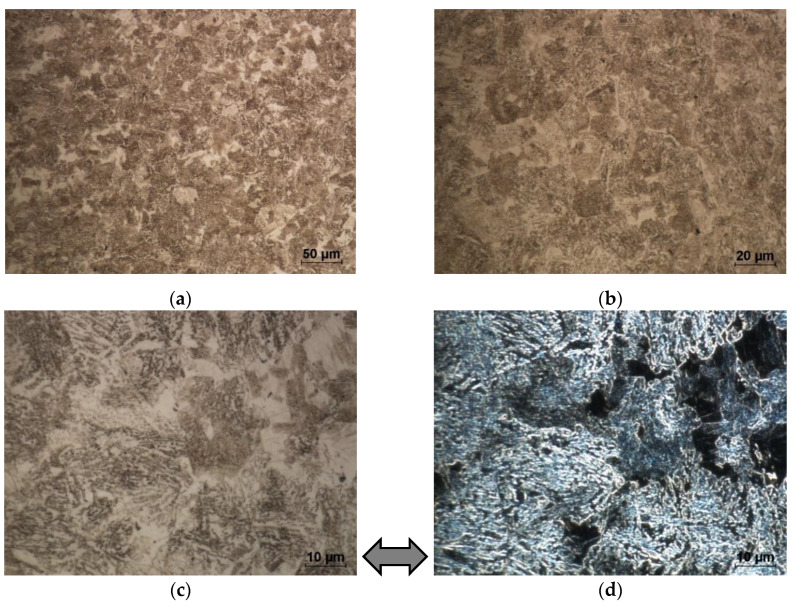
Microstructure of as-received 42CrMo4 steel (S0): (**a**–**c**) overview of the microstructure observed with different magnifications by bright field technique; (**d**) area from (**c**) photo observed by dark field technique (dark areas—ferrite, bright—cementite). Light optical microscopy (LOM), etched with Nital.

**Figure 3:**
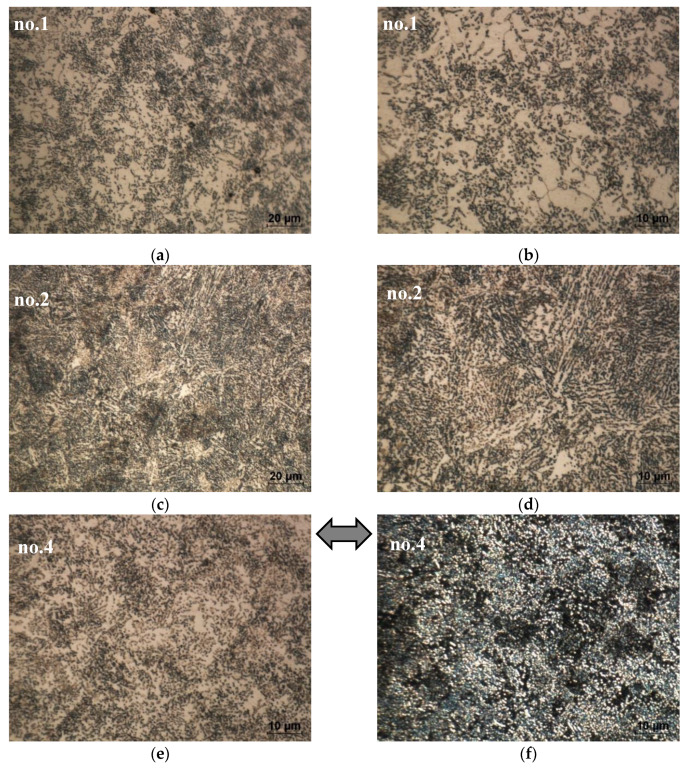
Microstructure of annealed 42CrMo4 steel with selected heat treatment schemes: (**a**–**e**) bright field technique; (**f**) area from (**e**) image captured by dark field technique (dark areas—ferrite, bright—cementite). LOM, 500× and 1000×, etched with Nital agent.

Microstructures of the steel before and after heat treatment are presented in Figure 2. The initial as-received 42CrMo4 coupon has a pearlite-ferrite microstructure (Figure 2) that is typical of hypoeutectoid steel [8,40,41]. Specifically, it can easily be observed in the dark-field image of the metallographic sample (Figure 2d) that the cementite (component of the pearlite phase) is visualised as a bright phase, while the ferrite areas are darker. Owing to the heat treatment, the alloy phase composition evolves, as shown in Figure 3. Moreover, the employed annealing scheme has a crucial effect on the 42CrMo4 microstructural development. 

**Figure 4:**
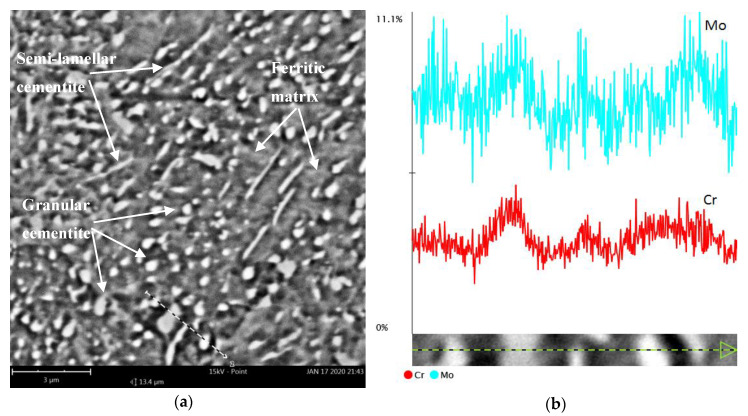
Microstructure of 42CrMo4 steel annealed according to scheme no. 4 (**a**), (**b**) molybdenum and chromium content in the line-scanned area from (**a**), scanning electron microscopy-energy dispersive spectrometer (SEM-EDS).

The metallographic examination confirms the phase development of the as-received steel microstructure (S0) (Figure 2) into a heat-treated microstructure; see Figure 3. The employed annealing schemes have a crucial effect on the microstructure’s morphology. The microstructure strongly develops owing to annealing and results in the evolution of cementite from lamellar to granular and semi-lamellar morphology. This process is less advanced for annealing schemes no. 2 and 3. Therefore, in the samples no. 1 and 4–7, the carbides are presented as semi-globular carbides in the ferritic matrix. The metallographic examination confirms that, in the as-received sample no. 0, the lamellar cementite has sharp edges, but after treatment, it undergoes rounding. The density of cementite decreases after annealing and so does the ferret diameter of the heat-treated cementite, that is, the mean ferret is 0.5 ± 0.25 µm, while the max ferret is 4.5 µm. A spheroidized microstructure is desirable for cold forming because it reduces the flow stress of the material [8]. In addition, in the bright-field image, one can easily identify the spheroid-like morphology of cementite (in comparison with the initial lamellar cementite in Figure 2). Moreover, it is clearly visible that the semi-globular cementite phase is enriched with alloying elements (such as Cr and Mo), which can result in the softening of a ferritic solid solution, as shown in Figure 4. This pearlite structure decomposition has a positive effect on the hardness decrease and deformability of steel, as discussed in further sections.

#### 3.1.2. Effects of Annealing Schemes on the Macro- and Microstructure of Upset Samples

**Figure 5:**
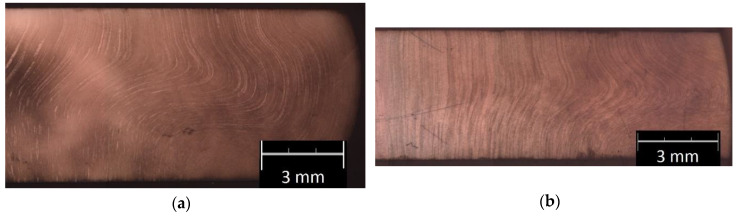
Transverse macrostructure of a half-view of the upset sample: (**a**) sample no. 0 and (**b**) annealed sample no. 6.

**Figure 6:**
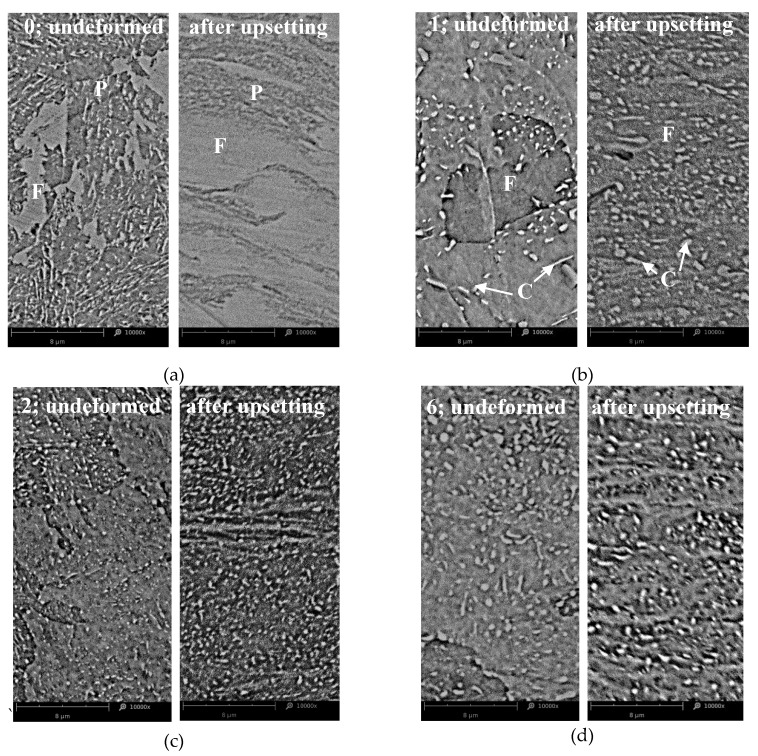
Effect of upsetting on the microstructure of 42CrMo4 steel samples: (**a**) as-received, (**b**) annealed according to scheme no. 1; (**c**) annealed according to scheme no. 2; (**d**) annealed according to scheme no. 6; P—pearlite, F—ferrite, C—cementite, SEM.

The employed annealing scheme affects the deformability of 42CrMo4 steel. Therefore, peripheral cracking occurs around the barrelled side surface of the upset (compressed) cylinders of the untreated sample, but no cracking occurs during compression of the annealed samples. Figure 5 shows the examples of the transverse macrostructure obtained by upsetting of the as-received sample (0) and the sample annealed according to scheme no. 6. The as-received sample exhibits a much wavier macroscale grain flow than the annealed cross section; see Figure 5. Therefore, the complex flow line arrangement confirms that the as-received steel has lower deformability than the annealed steel sample presented in Figure 5b. It is clear that annealing makes upsetting easier. Figure 6 compares the microstructural development owing to upsetting of the as-received and heat-treated steel. The annealed samples have less lamellar morphology of the cementite and the coarsening of granular tough-carbide phases and ferrite grains. Moreover, in relation to the as-received sample no. 0, they exhibit a higher rate of microstructure development. Contrary to the initial pearlite-ferrite microstructure of the 0 sample (Figure 6a), the granular cementite in the ferritic matrix of the heat-treated samples is beneficial for cold forming; see Figure 6b–d. As a result, there occurs a linear arrangement of the compressed annealed microstructure perpendicular to a direction of the compression force. The plastic flow of the material is facilitated by the lamellar to granular cementite development.

#### 3.1.3. Effect of Annealing on Phase Composition (XRD)

**Figure 7:**
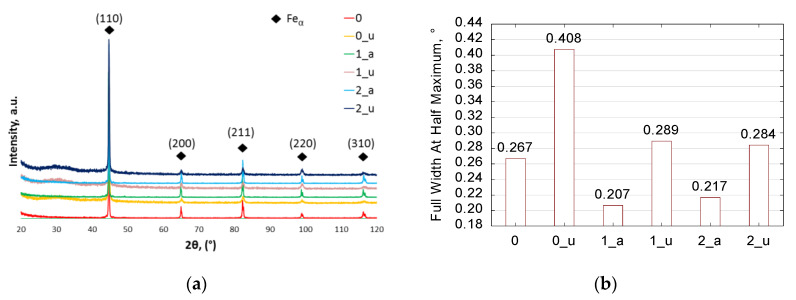
X-ray diffraction phase analysis of 42CrMo4 steel; (**a**) phase composition after annealing and upsetting; (**b**) quantitative microstructure phase analysis of an approximately 44° peak in the [110] plane; samples: 0—as-received; 1_a and 2_a—annealed; and 0_u, 1_u, and 2_u—upsetting; XRD.

X-ray diffraction phase analysis makes it possible to determine the phase composition of the investigated steel (Figure 7a) and the effects of heat treatment and upsetting on the microstructure composition of the samples (Figure 7b). An analysis of the diffractograms given in Figure 7a confirms that the steel has an α-Fe (ferritic) matrix. However, neither the cementite nor other carbides were identified by XRD, which can be explained by the dispersion of the cementite and carbides in the Fe-matrix, as well as the limited use of a Cu-lamp for XRD low-alloy steel carbide-phases detection. Nevertheless, the 44° peak broadening (Figure 7b) in the XRD analysis indicates that the heat treatment successfully affects the growth of ferrite grain size and relief of internal stresses. Compared with the 0 sample, the annealed samples no. 1_a and 2_a have a coarser microstructure; see Figure 7b. Usually, an increased grain size contributes to easier deformation. It is known that, especially for steels, the coarser grain size decreases mechanical properties such as hardness and yield strength, and can facilitate steel deformation [6,18,41,42]. Summing up, annealing scheme 1 is recommended for manufacturing cold-metal formed parts in the future [35,36].

In addition, the upsetting-induced grain size refinement was confirmed by the LOM and SEM-EDS results (presented in the previous section). The refinement by flattening of the ferritic grain matrix is identified in the deformed metallographic cross sections. This is in agreement with the obtained XRD quantitative data. Thus, the microstructural grain-size effects were confirmed by the XRD peak broadening, as shown in Figure 7b. The X-ray diffractogram results indicate that the calculated full width at half maximum (FWHM) for the upset samples presents wider peaks, which can be interpreted as refining of the grain size and increase of the internal stresses owing to cold-metal forming. This is in agreement with the fact that deformation usually provides a refined steel structure [40,43]. Thus, it can be seen that the SEM quantitative results are in agreement with the above observations, and it is clear that the upsetting process results in refinement of the microstructure.

### 3.2. Effect of Heat Treatment on Hardness

**Figure 8:**
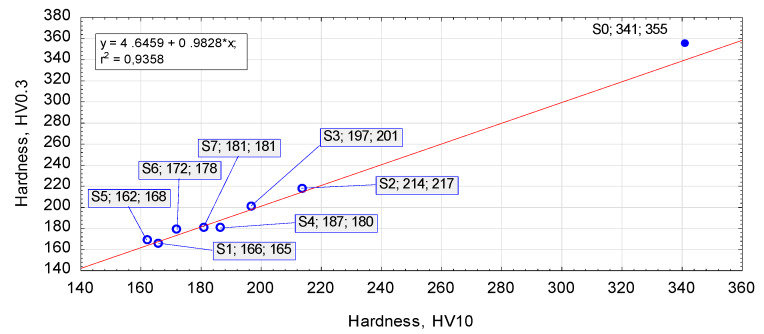
Hardness of as-received (S0) and annealed (S1–S7) samples.

**Figure 9:**
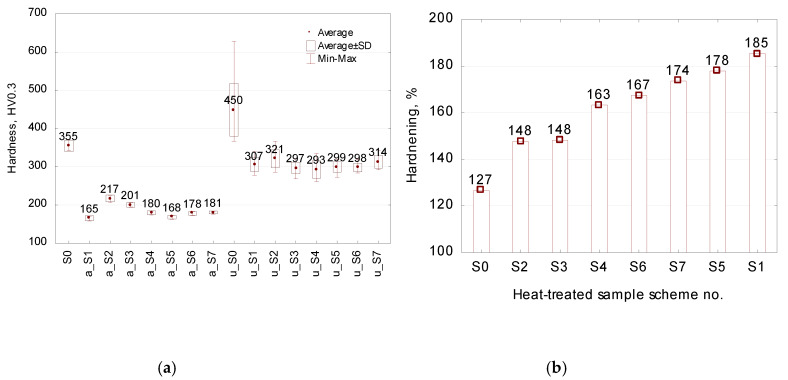
Hardness results of S0–S7 samples: (**a**) microhardness before and after upsetting (marked as “a” and “u”); (**b**) rate of cold hardening due to upsetting.

Hardness is the main indicator in quality assessment in a heat treatment. Figure 8 shows the effect of the employed annealing schemes on hardness. Specific hardness results and hardening rates are given in Figure 9. One can observe a visible linear correlation between the micro- and macro-hardness of the annealed samples. Every heat treatment scheme decreases the hardness of the as-received steel 4140 from approximately 350 HV to below 216 HV, which strongly facilitates deformability of the material. The annealing schemes no. 2 and 3 result in the hardness exceeding 200 HV and are considered as less effective. On the other hand, other investigated treatment schemes result in twofold lower hardness than that of the untreated steel sample no. 0. These results are in the range of hardness expected by the literature of the subject [8,9,15]. Summing up, the hardness results indicate that annealing scheme no. 1 has a more significant effect on increasing steel deformability than schemes no. 2 and 3 (see Figure 9b). These hardness results are in agreement with the FWHM findings and the results obtained by Bouras et al. [21], who observed a good correlation between the peak broadening parameter and the Vickers microhardness HV. Moreover, the above-mentioned development of lamellar cementite into a granular structure and the formation of a uniform α-Fe matrix are responsible for the decreased hardness and increased cold hardening rate, both of which are beneficial for increasing the formability of 42CrMo4 steel.

An analysis of the relationship between annealing and hardness (Figure 9) leads to the conclusion that scheme no. 1 has the best effect on steel formability. In addition, annealing schemes no. 1 and no. 2 (see Figure 1), which involve heating the material to the temperature of 750 °C followed by low-rate cooling in the furnace, yield lower hardness than schemes no. 4 and 5, which consist in heating the material to 750 °C and maintaining it at the temperature of 680 °C; furthermore, extension of the soaking time at 750 °C slightly affects the lowering of the hardness. What is more, the annealing scheme no. 6, that is, alternate heating and cooling, provides, in a shorter treatment time, hardness results that are comparable to those obtained with schemes no. 4 and 5. On the other hand, the prolonged holding of the material at the temperature just below A_1_ (schemes no. 2 and 3) does not provide satisfying results with regard to deformability of 42CrMo4 steel (Figure 9), which is why these parameters are not recommended. In addition, prolonging the cooling rate from 6 °C to 3 °C per hour (scheme no. 1 and no. 5, respectively) does not have any considerable effect on hardness, in spite of doubling the treatment time.

### 3.3. Analysis of Upsetting Test Results

**Figure 10:**
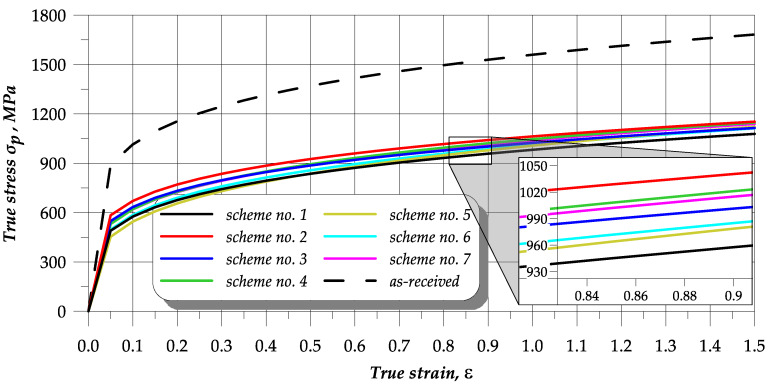
Upsetting flow curves of as-received (0) and annealed (schemes no. 1–7) 42CrMo4 steel.

The upsetting tests led to the determination of the *c* and *n* coefficients (given in Table 2) as well as the flow curves (plotted in Figure 10) for every heat treatment scheme. The results demonstrate that the flow stresses of the as-received samples are approximately 50% higher than those of the material in the annealed state. It can be seen that scheme no. 2 yields the highest stresses. Other annealing treatments result in decreasing the flow stresses required in cold forming. Thus, the microstructure of globular cementite in the ferritic matrix causes a reduction in the flow stresses in the upset samples. The lowest and the highest stresses are obtained for the annealing schemes 1 and 2, respectively. The difference between the stresses for the strain equal to, for example, 1, is about 80 MPa. Intermediate stress was obtained for scheme no. 3, where the difference between the stresses generated by the annealing schemes 1 and 2 is similar. The annealing process performed according to scheme no. 1 facilitates cold forming of the tested steel as it produces a structure of semi-globular carbides in the ferritic matrix that significantly affects the formability of this material.

**Figure 11:**
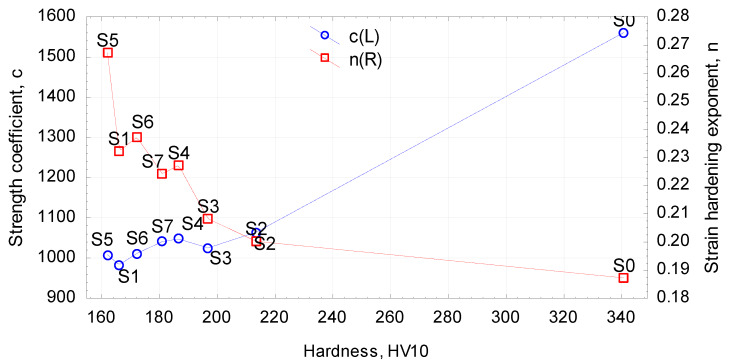
Effect of hardness on strain-hardening coefficients.

The effect of hardness of the samples on the strength coefficient *c* and the strain-hardening exponent *n* determined in the upsetting tests is presented in Figure 11. The annealed steel strength coefficient seems to increase with increasing hardness, while the strain hardening exponent decreases. However, there is no strong correlation between the hardness and the strength coefficient or the strain-hardening exponent. Summing up, it can be claimed that the treatment according to scheme no. 1 provides the most promising values of the *c* and *n* coefficients. Thus, this annealing scheme seems the most beneficial for cold metal forming of 42CrMo4 steel.

### 3.4. Comparison of the Numerical and Experimental Upsetting Results

**Figure 12:**
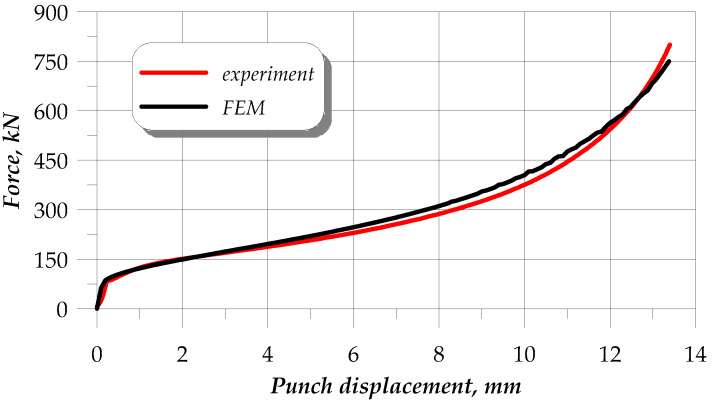
Finite element method (FEM) and experimental results of the upsetting force in scheme no. 3.

The determined flow curves were used in the numerical simulation of the upsetting of cylindrical samples, with the simulation conditions reflecting the experimental ones. The quality of the numerical results was assessed based on the force parameters. Figure 12 shows the force during the upsetting of samples that were annealed according to scheme no. 3, which is, as already mentioned, an intermediate scheme between extreme schemes no. 1 and 2. The experimental and FEM results show very high quantitative and qualitative agreement. In effect, the determined constitutive equations reflect well the real conditions. 

**Figure 13:**
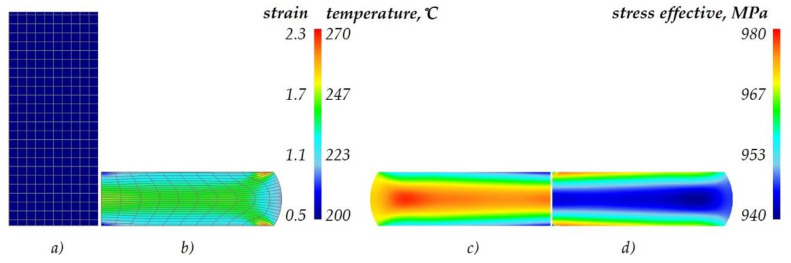
Numerical results of the upsetting process (half-view of the axial section): (**a**) start of the process, (**b**) effective strain, (**c**) temperature, (**d**) effective stress.

Selected numerical results are given in Figure 13. that show the distributions of effective strains and stresses as well as temperature. Owing to the fact that the flow curves of the material in the annealed state are similar, the distributions of the above-mentioned parameters are similar too. Consequently, the figure shows the results obtained for the flow curve determined in the upsetting of the samples annealed according to the intermediate scheme (no. 3). An analysis of the distribution of the effective strains (Figure 13b) that are presented together with a coordination mesh to show the lines of material flow reveals the presence of three typical and characteristic zones located at the end face, in the centre, and on the edge of the samples. The highest strains amount to approximately 2.3 and are located across the edge of the sample, right next to its end face. During the upsetting process, the temperature of the workpiece increased from 20 °C to about 270 °C. The highest temperature is observed in the central region of the samples (Figure 13c). The lowest temperature is observed on the end face of the sample, where the workpiece is in contact with the tools. As for effective strains (Figure 13d), the highest strains of approximately 980 MPa are located on the end face of the sample, while the lowest effective strains amounting to approximately 940 MPa are located in the central region of the sample. 

Calculated with the determined *c* and *n* coefficients, the flow lines and strains are in agreement with those reported in the literature of the subject [8,44]. Moreover, the simulation results given in Figure 13 were positively validated by the macroscale grain flow and the comparison of the experimental and FEM upsetting force-displacement results. This proves that the strain-hardening coefficients of the annealed samples can be used in FEM simulations for 42CrMo4 steel.

## 4. Conclusions

This study investigated the effects of annealing time and temperature on the microstructure, hardness, and strain-hardening coefficients of low-alloy structural steel grade 42CrMo4 (AISI 4140). In comparison with the as-received sample, all employed annealing processes improved the flow lines arrangement and facilitated microstructure softening, effectively decreasing the Vickers hardness and, consequently, enabling the formability of 42CrMo4 steel.

The results confirm that the optimal annealing treatment is scheme no. 1, consisting in maintaining the material at 750 °C and then slow-cooling it at the rate of 6 °C per hour. This scheme enables the cementite particles to attain the semi-globular morphology, which results in decreasing the hardness from 355 HV to 165 HV and obtaining the optimal strain hardening coefficients, that is, the strain coefficient and the strain hardening exponent are equal to c = 981 and n = 0.232, respectively. Moreover, the steel sample treated according to scheme no. 1 exhibits the highest cold hardening ability amounting to 185%, whereas that of the sample annealed according to scheme no. 2 is the lowest and amounts to 148%.

The formability of steel is affected by the adopted annealing scheme. The original strain hardening coefficients (*c* and *n*) for each of seven annealing schemes were determined. 

The results obtained by SEM-EDS, LOM, and XRD demonstrate that, owing to the employed treatment, the initial ferritic-pearlitic microstructure develops into granular and semi-lamellar precipitations of cementite enriched with Mo and Cr in the ferritic matrix. In addition, the annealing process affects the growth of α-Fe grains. These phenomena cause an almost twofold reduction in the hardness of the heat-treated steel and improve its cold-hardening properties.

The FEM results were positively validated by the experimental results of upsetting displacement versus force as well as microstructural investigations. This means that the calculated strain hardening coefficients can be used in numerical calculations when developing new metal forming methods for producing 42CrMo4 steel parts.

## Figures and Tables

**Table 1:** Nominal properties of 42CrMo4 steel.

Mechanical Properties, MPa		Chemical Composition ^1^, wt.%						
R_m_	R_e_	C	Mn	Si	P	S	Cr	Mo
1030	880	0.38–0.45	0.4–0.7	0.17–0.37	max 0.035	max 0.035	0.9–1.2	0.15–0.25

^1^ Ni max 0.3%; W max 0.2%; V max 0.05%; and Cu max 0.25%.

**Table 2:** Strain-hardening coefficients estimated for tested heat treatment schemes.

Strain Hardening Coefficient	Sample Code							
	0	1	2	3	4	5	6	7
**c**	1559.4	981.4	1062.7	1023.6	1045.8	1006.5	1009.9	1039.2
**n**	0.187	0.232	0.200	0.208	0.227	0.267	0.237	0.224
